# Emotion Regulation in the Preadolescent Brain and the Role of Individual Temperamental Differences

**DOI:** 10.1002/brb3.70895

**Published:** 2025-10-21

**Authors:** P. Cantou, B. Kleber, S. A. Kotz, P. Vuust, M. C. Fasano, E. Brattico

**Affiliations:** ^1^ Center For Music in the Brain, Department of Clinical Medicine Aarhus University & The Royal Academy of Music Aarhus/Aalborg Aarhus Denmark; ^2^ Faculty of Psychology and Neuroscience Department of Neuropsychology and Psychopharmacology Maastricht University Maastricht the Netherlands; ^3^ Department of Psychology and Behavioural Sciences Aarhus University Aarhus Denmark; ^4^ Department of Education Psychology, Communication University of Bari Bari Italy

## Abstract

**Purpose:**

This study investigated the association between emotion regulation, brain maturation, and self‐regulation traits in preadolescents, a developmental stage marked by substantial brain changes. An imbalance between hyperactive subcortical regions and an immature prefrontal cortex often leads to emotional instability and increased risk‐taking behaviors.

**Method:**

We conducted an event‐related functional magnetic resonance imaging (fMRI) study on preadolescents (*N* = 23; 10 females; mean age = 10.5 ± 1.3 years) using an emotional color flanker task to examine whole‐brain activation and seed‐based functional connectivity. Additionally, we assessed temperament traits to explore the relationship between neural correlates of emotional conflict resolution and self‐regulation abilities.

**Findings:**

Negative emotions impaired cognitive processing, particularly during conflict resolution. Preadolescents with stronger self‐regulation were quicker at resolving conflict under negative emotional conditions and showed reduced functional connectivity between cognitive‐emotional regions when processing negative versus neutral stimuli. Conversely, those with weaker self‐regulation showed heightened connectivity between the medial prefrontal cortex and ventral anterior insula when processing negative words.

**Conclusion:**

Our findings underscore the role of individual differences in brain connectivity and temperamental traits in emotion regulation during preadolescence. Enhanced self‐regulation is linked to more efficient emotion processing and distinct neural connectivity patterns, highlighting the importance of incorporating neurobiological and temperamental factors in developmental studies of emotion regulation.

## Graphical abstract

Here, we examined the link between emotion regulation in the brain and individual self‐regulation traits in preadolescence by using a color flanker task in a functional magnetic resonance imaging (fMRI) setting. Results revealed that preadolescents with better self‐regulation were faster to resolve conflict in a negative emotional situation (better emotion regulation) and exhibited lower functional brain connectivity between cognitive‐emotional areas (anterior insula and medial prefrontal cortex) when processing negative vs. neutral stimuli than those with poorer self‐regulation. These findings underline the role of individual cerebral and temperamental differences in emotion regulation in preadolescence.

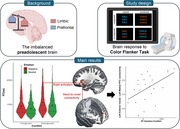

## 1 Introduction

Adolescence and its immediately preceding period termed preadolescence are transitional phases between childhood and adulthood characterized by major physical and behavioral changes such as biological hormonal changes, neurological reorganization, and increased sensitivity to social and emotional information (Spielberg et al. 2014). This challenging transition also involves the intake of complex information, increased academic demands, as well as dealing with reward sensitivity, emotional instability, risk‐taking, and impulsive decision‐making (see Shulman et al. [2016] for a review).

As preadolescents and adolescents gradually gain independence from their parents, emotion regulation needs to increase in order to cope with emotional situations (Young et al. 2019). Furthermore, adaptive emotional regulation is necessary for situational social adjustments where preadolescents and adolescents increasingly seek a peers’ acceptance, but also for mental health, as adolescence is a high‐risk period for the emergence of psychological disorders such as depression and anxiety (Balázs et al. 2013). These disorders are characterized by exaggerated emotional reactivity to negative cues and difficulty in downregulating this response (Lennarz et al. 2019). This suggests that effective regulatory processes are necessary to maintain a healthy psychological state. However, these behavioral changes are also a normal step in development, allowing adolescents to build up new strategies for managing emotions and self‐sufficiency (Garnefski et al. 2005). Cognitive processes such as attention, response inhibition, and executive control contribute to emotional regulation (Schmeichel and Tang 2015), but the degree to which cognition and emotional processing interact in this developmentally sensitive period remains unclear (Fasano et al. 2023).

To better understand these dynamic changes, neuroimaging studies investigated emotion processing across the lifespan, focusing on brain areas involved in controlling both emotion and cognition, such as the amygdala, the dorsal medial prefrontal cortex (mPFC), and the lateral prefrontal cortex (lPFC). The amygdala displays an age‐related decline in activity in response to negative stimuli, although results are mixed as to whether its reactivity peaks during late childhood, preadolescence, or adolescence (Hare et al. 2008; Gee et al. 2013; Vink et al. 2014; Silvers et al. 2017). Findings regarding prefrontal cortex (PFC) activation are also inconsistent. Some reported that adults exhibit greater PFC activation than adolescents in emotional regulation tasks (Casey et al. 2015; Shulman et al. 2016), while others found that adolescents show greater PFC activation than children or adults (Crone and Dahl 2012; Pfeifer and Allen 2012). Such discrepancies highlight the need to go beyond localized activation changes in favor of connectivity between frontal and limbic regions.

Several studies addressed connectivity in emotional regulation tasks and reported a developmental switch from positive to negative functional connectivity between the amygdala and the mPFC during the transition from late childhood to preadolescence (Gee et al. 2013; Wu et al. 2016). More recently, it has been shown that age predicts reduced amygdala activity via increased recruitment of the ventral lPFC during reappraisal, associated with individual levels of maturation of mPFC–amygdala connectivity, that is, more negative connectivity as a function of age (Silvers et al. 2017).

Building on these findings, emotion regulation has been shown to rely on dynamic interactions between the PFC and limbic regions such as the amygdala, with functional connectivity between these regions undergoing substantial developmental changes from childhood through adolescence. Notably, prior studies have identified a shift from positive to inverse amygdala–PFC connectivity across this trajectory (Gee et al. 2013; Wu et al. 2016), interpreted as increasing top‐down control of emotional reactivity (Casey et al. 2019).

Preadolescence represents a unique and understudied window in this transition, marked by increasing cognitive control abilities, but still‐maturing regulatory brain systems (Somerville et al. 2010; Pfeifer and Allen 2012). Despite its theoretical importance to understand emotion regulation, only a few neuroimaging studies have focused on this phase (Fasano et al. 2023), resulting in a gap in understanding how fronto‐limbic connectivity supports emerging emotion regulation before adolescence proper.

While much of the existing work has emphasized group‐level age effects, individual differences, such as trait negative affect or effortful control, may modulate these neural dynamics in ways that shape developmental trajectories but remain largely unexplored at this age (Davis et al. 2019). Investigating preadolescents can therefore offer critical insight into the early neural architecture of emotion regulation and the factors that may predict adaptive or maladaptive outcomes.

These results are consistent with the neurobiological imbalance model (see Casey et al. [2019] for a review), which proposes that emotional fluctuations in adolescence are due to connectivity changes from subcortical to cortical circuits. This model is based on prior human and rodent neuroimaging studies, showing that projections from the PFC to the limbic system mature throughout adolescence and are associated with enhanced cognitive control in emotional situations (see Caballero et al. [2016] for a review).

However, the ability to regulate one's emotions varies widely among individuals, and most studies focused on age‐related changes without providing a concise account of how interactions between the PFC and the limbic system are altered by interindividual differences.

Few studies investigated interindividual differences and found that adolescents high in trait anxiety exhibit less amygdala habituation after repeated exposure to emotional faces (Hare et al. 2008). In adults, more negative amygdala–PFC connectivity in individuals with less pupil dilation and better facial muscle control was reported. These are indicators of effective emotion regulation (Lee et al. 2012). Interestingly, a recent study focused on two temperament traits when examining emotional regulation: negative emotionality and cognitive control (Davis et al. 2019). The authors used an emotion regulation task and found that negative emotionality predicts a less mature pattern of amygdala–lPFC connectivity (more coupling), whereas cognitive control predicts a more mature pattern (less coupling). These results highlight the need to specify the relationship between individual differences in emotional regulation and fronto‐limbic connectivity to prevent the development of maladaptive strategies in adolescence.

The present study investigated individual differences and the neural correlates of emotional regulation in preadolescents using functional magnetic resonance imaging (fMRI). For evaluating the effect of negative emotions on cognitive control, we adapted the color flanker task (CFT) originally proposed for German adults (Kanske and Kotz 2012) for Danish preadolescents. Moreover, we administered a temperament trait questionnaire, assessing negative affect (sensitivity to negative emotions) and effortful control (general cognitive abilities) (Ellis and Rothbart 1999). We hypothesized that trials of negative valence (later on referred to as negative trials) would (1) modulate cognitive control as reflected in reaction times (RT), (2) increase brain activity in emotion‐responsive areas such as the amygdala, (3) enhance amygdala–PFC functional connectivity, and (4) be associated with higher amygdala–PFC connectivity in individuals with lower self‐regulation abilities (high negative affect and low effortful control).

## 2 Methods

### 2.1 Participants

Participants were part of a longitudinal study addressing the impact of musical training on self‐regulation abilities and brain plasticity (Cantou et al., in preparation). At the time of this study, none of the children had received any formal music training. The music group was recruited from the waiting list of Aarhus Music School and had not yet started instrumental instruction. The control group was recruited through Facebook advertisements. Both groups attended regular public schools in the Aarhus areas. The current study was conducted at the pretraining stage, prior to the onset of any music lessons.

A total of 26 neurotypical preadolescents (8.5–12.5 years old) participated in the experiment. This age range was selected based on widely accepted developmental definitions of preadolescence as the transitional period preceding adolescence, typically spanning from late childhood into early puberty. It is a time marked by emerging neurobiological, cognitive, and emotional regulation changes, often beginning before visible signs of puberty (Dahl et al. 2018; Nook et al. 2019). Similar age ranges have been used in previous neuroimaging studies targeting emotion–cognition interactions during this phase (e.g., Gee et al. 2013; Raschle et al. 2017). Pubertal status was assessed using the Pubertal Development Scale (PDS; Petersen et al. 1988), with puberty categories computed following the criteria described by Crockett (1988, unpublished; see sleepforscience.org). Most participants were between prepubertal and midpubertal stages, with two girls reaching postpubertal status (see Table S1). Three participants were excluded from the analyses: one because of excessive movement artifacts, one due to failure to complete the entire task (i.e., inability to tolerate the scanning environment), and one showed an abnormal structural MRI. Accordingly, we included 23 participants (10 girls) in the analyses with a mean age of 10.5 (SD 1.3). All participants were native Danish speakers, right‐handed, could read and write, had normal or corrected‐to‐normal vision, had not been diagnosed with a developmental or psychiatric disorder or been prescribed psychotropic medication, and had no medical conditions contraindicated for scanning. Written informed consent for participation was obtained from the parents or guardians on behalf of the preadolescents, and verbal assent was obtained from all children. Both parents and preadolescents could quit the study at any time. They received voucher compensation for their participation in the form of cinema tickets, Amazon vouchers, and small toys for the participants. All procedures were approved by the Central Denmark Region Science Ethics Committee (prot. n. 1‐10‐72‐444‐17).

### 2.2 Experimental Procedure

All preadolescents were tested individually at the MindLab facilities of the Center of Functionally Integrative Neuroscience (CFIN) located at Aarhus University Hospital. For the scanning session, we designed a child‐friendly protocol that included a 20‐min preparation prior to the actual scanning. Preadolescents were carefully informed about the functioning of the MRI, what they would do during scanning, and how important it was to stay still, with the help of some pictures. They also had some time to explore the scanner room and test the bed for a brief familiarization period. To avoid any distress for the individual child, the parents were given the possibility to accompany the child during preparation in the MR room. The scanning time was 43 min and included four different sequences: resting‐state (8 min), CFT (12 min), a brief musical excerpt (4 min), and an anatomical sequence (MP2RAGE; 11 min) during which participants watched the cartoon “The Lego Story” available on YouTube. Psychological tests and questionnaires were administered after the MRI session in a quiet behavioral testing room with the assistance of a Danish psychology student, previously trained and supervised by a licensed psychologist.

### 2.3 Questionnaire for Assessing Individual Temperamental Traits

Our preadolescent participants and their parents completed the revised short form of the Early‐Adolescents‐Temperament‐Questionnaire (EATQ‐R) to assess their individual differences in self‐regulation abilities (Ellis and Rothbart 1999). We selected effortful control and negative affect, which are two higher‐order super scales composed of multiple subscales. Specifically, effortful control (16 preadolescent‐report items, 19 parent‐report items) includes the subscales inhibitory control, activation control, and attentional control, while negative affect (19 child‐report items, 18 parent‐report items) includes frustration, depressive mood, and aggression. In the present study, only the super scales were analyzed to focus on general cognitive abilities and sensitivity to negative stimuli. Preadolescents were instructed to judge how well an item described them, and parents were instructed to evaluate how well an item described their child. There were five response options ranging from “almost always untrue” to “almost always true.” Scores of 1–5 were assigned to each response option, and mean scores for each participant and for each form (preadolescents and parents) were calculated. Example items from the preadolescent's report form for effortful control were as follows: “It's hard for me not to open presents before I'm supposed to,” “It is easy for me to really concentrate on homework problems,” and “If I have a hard assignment to do, I get started right away.” Example items for negative affect were as follows: “My friends seem to enjoy themselves more than I do,” “When I am angry, I throw or break things,” and “I get very frustrated when I make a mistake in my schoolwork.” Items from the parent's report form were similar.

### 2.4 Color Flanker Task

## 3 Experiment

In the MRI scanner, participants performed a Danish adaptation of the CFT, originally developed for German adult participants (Kanske and Kotz 2011). Words were selected from the Leipzig Affective Norms for German (Kanske and Kotz 2011) and translated into Danish. The Danish translations were matched for letter and syllable length, and their arousal (degree of emotional intensity) and valence (positive or negative emotional value) ratings were confirmed using a 9‐point Likert scale with an independent, age‐matched group of 18 preadolescents (see Tables 1 and 2).

**TABLE 1:** Number of letters and syllables for the negative and neutral words in German and Danish languages. Corresponding ratings of valence and arousal were acquired in an independent sample using a 9‐point Likert scale.

Word type	Example word (German)	Example word (Danish)	Number of letters (German)	Number of letters (Danish)	Number of syllables (German)	Number of syllables (Danish)	Rated valence	Rated arousal
Negative	Tod	Død	5.50 (1.06)	5.65 (1.86)	1.88 (0.64)	1.95 (0.9)	2.05 (0.87)	7.55 (0.48)	
Neutral	Phase	Fase	5.87 (1.09)	5.95 (1.75)	2.00 (0.39)	1.95 (0.71)	4.97 (0.19)	2.48 (0.67)

**TABLE 2:** Statistical differences in the number of letters/syllables, rated valence/arousal for negative and neutral words.

	*t*‐value	*p*
Number of letters (negative; German–Danish)	−0.44	0.66
Number of letters (neutral; German–Danish)	−0.23	0.82
Number of syllables (negative; German–Danish)	−0.42	0.68
Number of syllables (neutral; German–Danish)	0.33	0.74
Rated valence (Danish; negative–neutral)	−26.57	< 0.001
Rated arousal (Danish; negative–neutral)	36.73	< 0.001

**FIGURE 1:**
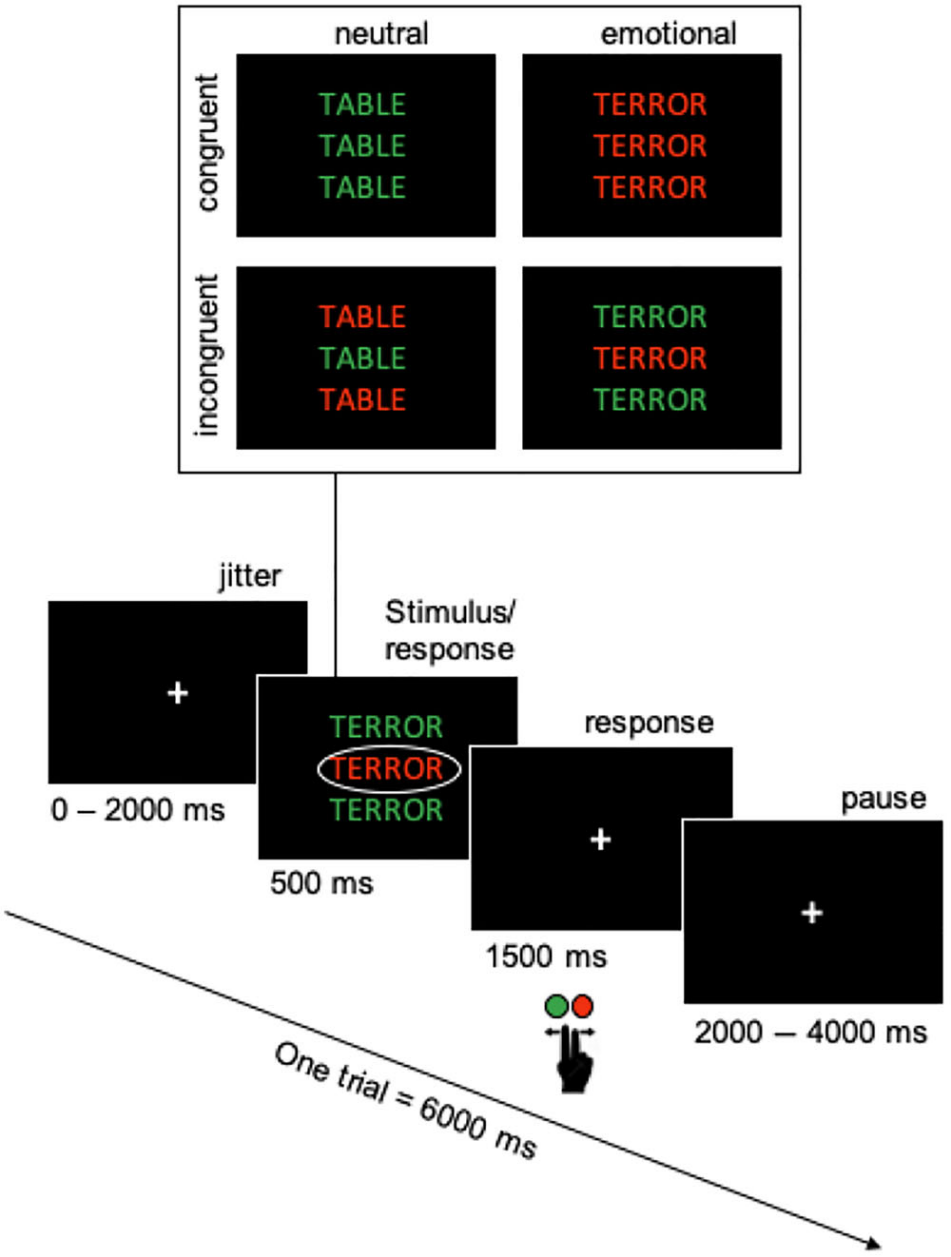
Color flanker task used in this study. The participant's task was to name the print color of the center word, ignoring that of the flanker words (the actual colors were red and green). Words were either emotional or neutral, yielding a fully crossed design of conflict and emotion (adapted from Kotz and Kanske 2011).

The CFT consisted of the presentation of three words on a black screen, of which participants had to identify the ink color of the middle word while ignoring the color of the flanker words above and below the target word (Figure 1). Participants were given a button box in their right hand to press the corresponding button to the target word color using their index and middle fingers. Flanker and target colors could be either identical or different, creating congruent and incongruent trials. Half of the words were of neutral valence, and the other half were of negative valence in order to study the influence of negative emotion on the resolution of response conflict in incongruent trials. A total of 80 words (20 per condition) were presented, crossing emotional valence (negative vs. neutral) and conflict (congruent vs. incongruent). Each word appeared once, and colors were pseudorandomly assigned to the 80 trials such that emotional and neutral words were presented equally often in green or red. To avoid confounding effects of color, this color assignment was fully counterbalanced across participants and conditions, ensuring that color was not systematically associated with emotion or conflict conditions. All trials were presented in pseudorandomized order, meaning that trials were randomized for each participant while ensuring an even distribution of conditions across the task. The presentation time was 500 ms, and the maximal response time was 2000 ms. Each of the 80 trials lasted 6000 ms. At the start of each trial, a fixation cross was presented for a jittered duration (0–2000 ms) to avoid temporal orienting, followed by the stimulus (500 ms). Participants were expected to respond within 2000 ms after stimulus onset. However, to avoid data loss from slightly delayed responses observed during early testing, responses were recorded throughout the remaining poststimulus interval. This poststimulus period, which also served as a fixation pause before the next trial, varied in duration depending on the initial jitter, ranging from 3500 ms (when jitter = 2000 ms) to 5500 ms (when jitter = 0 ms). The experiment lasted about 12 min, including about 4 min of no‐stimulation baseline, evenly distributed across the two scanning blocks. Stimuli were presented with the Presentation software. After instructions, participants completed a practice block of 16 trials followed by two separated blocks of 40 trials.

### 3.1 RTs and Accuracy

We used R (R3.6.0, GUI 1.70) and the function *lmer* from the *lme4* (Bates et al. 2014) package to perform a linear mixed effects analysis testing whether RT was dependent on CONFLICT (congruent/incongruent) and EMOTION (neutral/negative). In contrast to the more traditional repeated‐measures ANOVA analysis, this analysis allows controlling for the variance associated with random factors (Winter 2013). We created four different models, from the simplest to the most complex, and performed a likelihood ratio test to determine which one would best explain the data. The null model (Model1) only included the PARTICIPANTS variable as a random effect, then the factor CONFLICT was added in the second model (Model2), and the factor EMOTION was added in the third model (Model3) and in the fourth model with an interaction term (Model4). CONFLICT and EMOTION were always entered as fixed effects, whereas PARTICIPANTS was always treated as random effects. *p*‐values were obtained by likelihood ratio test using an ANOVA by comparing more complex models with simpler ones in order to assess the full model (with the effects of interest) against the reduced model (without the effects of interest). Following the latest best practice guidelines for linear mixed‐effect models in psychological science, we used the Akaike information criteria (AIC) for model comparisons (Meteyard and Davies 2020) and selected the model with the smallest AIC (Supporting Information; Table 2) to determine significant main effects and interactions (Table 3). Based on significant main effects, we finally computed Bonferroni‐corrected post hoc pairwise comparisons between the conditions using least square means. Note that each word was presented only once across the entire task, without repetition across conditions. Given this design, item‐level variability could not be estimated, and the item was therefore not included as a random factor in the linear mixed‐effects model. All words were carefully matched for linguistic and affective properties (see Table 1), and conditions were fully counterbalanced to minimize item‐related confounds.

**TABLE 3:** Linear mixed model fit by maximum likelihood of the fixed and random effects of Model3 for reaction time.

FIXED EFFECTS			
	ESTIMATE	SE	*ttt*‐value
(Intercept)	9268.0	163.7	56.6
CONFLICT (incongruent)	1552.1	231.6	6.7
EMOTION (negative)	915.4	231.6	3.96
**RANDOM EFFECTS**			
**GROUPS**	**NAME**	**VARIANCE**	**SD**
PARTICIPANTS	(Intercept)	7,383,641	2717
RESIDUAL		5,706,035	2389

*Note*: CONFLICT and EMOTION were coded with “congruent” and “negative” as reference levels.

Abbreviations: SD = standard deviation, SE = standard error.

To obtain a measure for conflict processing speed, we used the RT difference between incongruent and congruent trials, later referred to as *conflict score*. A higher score represents slower conflict resolution. As a measure of the influence of emotion on conflict processing, we used the difference between conflict scores in emotional and neutral trials, referred to as *emotion‐conflict scores*. This yields higher scores when conflict processing is slower in emotional trials than in neutral trials. In other words, an emotion‐conflict score reflects the ability to resolve conflict in an emotional condition versus the ability to resolve conflict in a neutral condition.

To investigate individual differences in conflict and emotional processing, we performed Pearson's correlations between both conflict and emotion‐conflict scores with negative affect and effortful control scores (Tables 3 and 4). To correct for multiple comparisons, we used the Benjamini–Hochberg method (Benjamini and Hochberg 1995), which controls the false discovery.

**TABLE 4:** Least squares mean comparisons for Model3 for reaction time. *p*‐values are adjusted using Bonferroni correction.

	Contrast	ESTIMATE	SE	t.ratio	*p*‐value	Cohen's *d*
EMOTION	Negative–neutral	1049	103	10.14	< 0.0001	0.29
CONFLICT	Incongruent–congruent	1418	103	13.71	< 0.0001	0.40

Abbreviation: SE = standard error.

To assess accuracy, we performed a generalized linear mixed model (GLMM) analysis using the *glmer* function from the lme4 package. Accuracy was modeled as a binary outcome (correct/incorrect) with a binomial distribution and logit link function. The model included CONFLICT (congruent/incongruent) and EMOTION (negative/neutral) as fixed effects, and PARTICIPANTS as a random intercept to account for individual differences.

#### 3.1.1 fMRI Processing and Analyses

#### 3.1.2 Data Acquisition and Preprocessing

Neuroimaging data were collected at 3T (Siemens Prisma) with a 32‐channel head coil. To restrict head motion and attenuate scanner noise, foam cushions were placed around the head and arms. The participants wore headphones to listen to a cartoon projected on a screen during the anatomical sequence. The CFT was presented using Presentation software (https://www.neurobs.com/). BOLD‐weighted fMRI data were acquired using a gradient echo‐planar imaging (EPI) multiband sequence (TR = 1000 ms; TE = 29.6 ms; voxel size = 2.5 mm^3^). High‐resolution T1‐weighted images (TR = 6500 ms; TE = 3.47 ms; voxel size = 0.9 mm^3^) were obtained using an MP2RAGE sequence, during which preadolescents watched a short cartoon to attenuate movement artifacts.

The fMRI data were preprocessed with SPM12 (https://www.fil.ion.ucl.ac.uk/spm/) implemented in Matlab R2016b (Mathworks), FSL (www.fmrib.ox.ac.uk/fsl) using the MELODIC tool (version 3.14) and FIACH (Functional Image Artefact Correction Heuristic) (Tierney et al. 2016) implemented in R (R3.6.0, GUI 1.70).

1. In SPM12, functional EPI data of each participant were realigned to the first image of the session to correct for head motion, and high‐resolution anatomical images of individual subjects were then co‐registered to the mean functional EPI.
2. The realigned images were preprocessed with FIACH to remove nonphysiological signal changes. The six FIACH noise regressors were later entered as confounds in the analysis.
3. An additional noise removal method was applied to the FIACH‐preprocessed functional EPI with MELODIC‐ICA in FSL. This method uses probabilistic spatial independent component analysis (ICA), decomposing each participant's fMRI session into *N*_components_ × time, with the number of components, *N*_components_, being estimated automatically through Bayesian dimensionality estimation techniques (Beckmann and Smith 2004). Each component was represented by a spatial map and a time course. The resulting ICA decompositions (one per participant) were reviewed independently and classified into noise‐ and signal‐based (Griffanti et al. 2017) for the frequency content and appearance of the time course and for the pattern of the spatial map of each component. Following this hand classification, the FSL function *fsl_regfilt* was used to recompose the data to *N*_voxels_ × time while regressing out the contribution of the components labeled as noise.
4. The resulting functional EPI was normalized in SPM12 after entering the tissue probability maps of an average brain template in MNI space for young participants aged between 7.5 and 13.5 years (Fonov et al. 2011) and spatially smoothed by means of an isotropic Gaussian kernel of 6 mm full‐width at half‐maximum (FWHM).

#### 3.1.3 Data Analysis

A general linear model (GLM; Friston et al. 1995) was created in SPM12 for each participant's data using regressors that corresponded to the four task conditions: negative, congruent; negative, incongruent; neutral, congruent; and neutral, incongruent. High‐pass temporal filtering with a cutoff of 128 s was applied to remove low‐frequency drift in the data. Using an event‐related design, we convolved the hemodynamic response with the onset of each experimental event, while accounting for motion artifacts using the six FIACH realignment parameters as regressors of no interest. We created contrast images for every participant by comparing each condition with the no‐stimulation baseline, which was implicitly modeled in SPM at the first level. These contrasts were entered into the second‐level model using a full factorial design with EMOTION and CONFLICT as factors, representing each of the two levels (neutral/negative, congruent/incongruent).

We conducted whole‐brain analyses to investigate the main and interaction task effects in the entire group and employed cluster‐extent‐based thresholding using Monte Carlo simulation to correct for multiple comparisons (Zamorano et al. 2019) as implemented in DPABI's instantiation of AlphaSim (Cox 1996; Yan et al. 2016). Cluster‐extent significance thresholds were set at *p* < 0.05 FWER correction for multiple comparisons, followed by a voxel‐level threshold of *p* < 0.001. Only clusters surviving the FWER probability threshold were used for statistical inference (cluster size > 30 voxels). Subsequently, we conducted seed‐based connectivity analysis using the CONN toolbox, version 18.b (http://www.nitrc.org/projects/conn; Whitfield‐Gabrieli and Nieto‐Castanon 2012). To further investigate emotion regulation neural processes, we investigated the connectivity between regions found as functionally involved in the main effect of EMOTION from the GLM group analysis, that is, the left ventral anterior insula (lvAI), the right superior frontal gyrus (rSFG), and the right anterior cingulate cortex (rACC). We defined these three regions as regions of interest (ROIs). Given the central role of the vAI in affective processing (Uddin et al. 2017), a particular focus on the seed‐based connectivity from this region was made in the results section. The ROIs were extracted from the significant activation clusters using Marsbar (Brett et al. 2002), a software for ROI analysis of SPM data (marsbar.sourceforge.net). All the thresholded activation clusters were saved as a single binary image in the Montreal Neurological Institute (MNI) standard anatomical space used by SPM. Based on the previous literature, we also added bilateral amygdala as ROIs based on the AAL atlas as implemented in CONN.

Correlation coefficients were calculated for each subject by extracting the BOLD time course from each ROI and then by computing the correlation coefficient between that and the time courses from all other brain voxels during each of the four conditions. Correlation maps were produced based on this computation. Second‐level seed‐to‐voxel analyses were then completed to allow for group‐level comparisons. As in the GLM analysis, we conducted a full factorial design using the factors EMOTION (neutral/negative) and CONFLICT (congruent/incongruent). Significance thresholds were again estimated via Monte Carlo simulations using the resultant F‐maps. Cluster‐extent significance thresholds were set at *p* < 0.05 FWER correction for multiple comparisons, followed by a voxel‐level threshold of *p* < 0.001. Only clusters surviving the FWER probability threshold (cluster size > 30).

Individual‐level beta values (Fisher's *Z*‐transformed correlation coefficients) for clusters identified at this significance threshold were extracted and entered into R to perform Pearson's correlation with the behavioral scores (negative affect, effortful control, conflict score, and emotion‐conflict score).

## 4 Results

### 4.1 Behavioral Data

#### 4.1.1 RT and Accuracy

We removed all response latencies above 3000 ms from the analysis, which could have resulted from the participant getting bored, distracted, or forgetting which button to press. In total, 3.6% of the trials were excluded.

RT was analyzed at the trial level using linear mixed‐effects models. To assess the normality assumption, we examined residuals from the RT model. *Q*–*Q* plots showed positive skewness, and a Shapiro–Wilk test confirmed deviation from normality (*W* = 0.92, *p* < 0.001). We reran the model using log‐transformed RTs, which yielded consistent results. For interpretability, we report the raw RT model below (see Tables S3 and S4 for the log‐transformed version).

As described above, the individual models with significant effects were compared using an ANOVA‐based likelihood ratio test with a chi‐square test (Supporting Information; Table 2). The winning model (Model3) included a main effect of CONFLICT and EMOTION (Table 3), and the likelihood ratio test found no significant increase in model fit with the addition of the interaction factor (Model4). These findings indicate that the CONFLICT × EMOTION interaction does not contribute significantly to variance beyond the main effects. As a result, CONFLICT and EMOTION appear to have independent effects on RT. The winning Model3 showed that RT was higher in the incongruent condition (by 1552.1 ms) than in the congruent condition and was higher in the negative condition (by 915.4 ms). The model accounted for 5.9% of the variance through fixed effects (R^2^m) and 62.4% including random effects (R^2^c), indicating substantial individual variability. The pairwise comparisons (Table 4) confirmed that RT for the incongruent and negative conditions was significantly higher than for the congruent and neutral conditions. Effect sizes calculated from pairwise comparisons yielded Cohen's *d* values of 0.40 for CONFLICT and 0.29 for EMOTION, indicating moderate and small‐to‐moderate effects, respectively.

Accuracy was analyzed at the trial level using generalized linear mixed models (GLMMs) with a binomial distribution. As with the RT analysis, four models were compared using ANOVA‐based likelihood ratio tests (see Supporting Information; Table 5). The full model including the main effects of CONFLICT and EMOTION and their interaction showed the best fit (Model4), though the improvement over the additive model was not significant (*p* = 0.10). None of the fixed effects reached significance: CONFLICT (*z* = −0.64, *p* = 0.52), EMOTION (*z* = −0.22, *p* = 0.83), or their interaction (*z* = −1.65, *p* = 0.10). Pairwise comparisons were also conducted but did not reveal any significant differences after Bonferroni correction (see Supporting Information; Table 6), including the contrast between negative incongruent and neutral incongruent (*z* = −2.48, *p* = 0.079).

**TABLE 5:** General linear mixed model fit by maximum likelihood of the fixed and random effects of Model4 for accuracy.

FIXED EFFECTS					
	ESTIMATE	SE	*z*‐value	*p*	
(Intercept)	2.546	0.330	7.707	< 0.001	
CONFLICT (incongruent)	−0.134	0.208	−0.643	0.520	
EMOTION (negative)	−0.047	0.214	−0.220	0.826	
CONFLICT × EMOTION	−0.511	0.310	−1.645	0.100	
**RANDOM EFFECTS**					
**GROUPS**	**NAME**	**VARIANCE**	**SD**		
PARTICIPANTS	(Intercept)	1.763	1.328		

*Note*: CONFLICT and EMOTION were coded with “congruent” and “negative” as reference levels.

Abbreviations: SD = standard deviation, SE = standard error.

**TABLE 6:** Pearson's correlations between effortful control and negative affect from both parents and preadolescents.

		Effortful control (parents)	Effortful control (preadolescents)
Negative affect (parents)	puncorr	0.005**	
	pcorr	0.01**	
	*R*	−0.56	
Negative affect (preadolescents)	puncorr		0.11
	pcorr		0.11
	*R*		−0.34

*Note*: puncorr represents the *p*‐values uncorrected for multiple comparisons; pcorr represents the *p*‐values corrected for multiple comparisons using the Benjamini–Hochberg method.

**p ≤ 0.01.

#### 4.1.2 Correlations Between Temperamental Traits and Behavioral Responses

**FIGURE 2:**
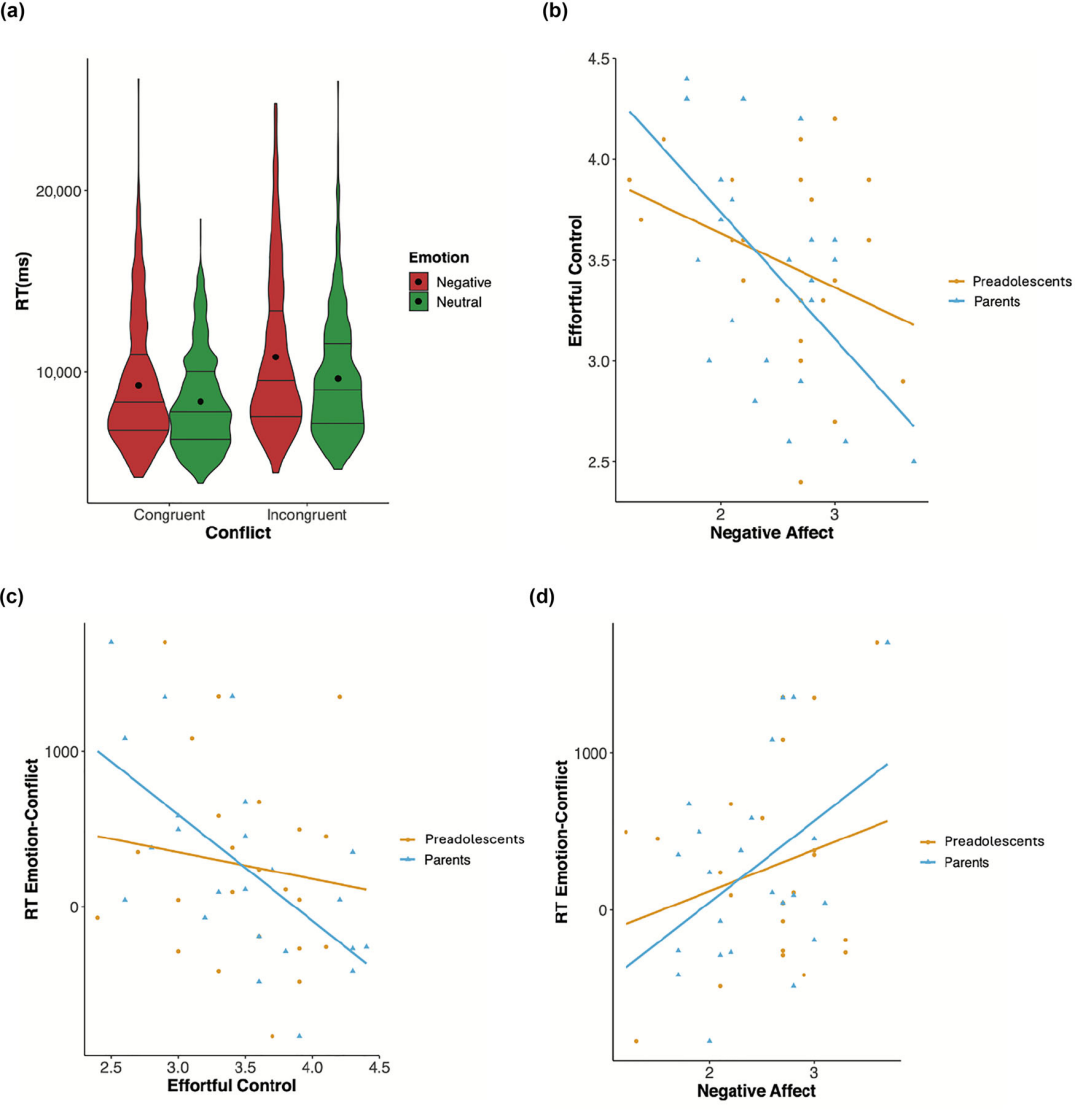
(a) Mean reaction times and distribution for all participants and the four conditions. (b–d) Pearson's correlations graphs between individual effortful control and negative affect scores from both parents and preadolescents, and between these two temperament traits and individual emotion‐conflict score.

We correlated individual data on effortful control with negative affect from both parents and preadolescents to establish a relationship between these two temperamental traits (Figure 2b; Table 6). This showed a significant negative correlation only for effortful control and negative affect evaluated by the parents (*R* = −0.56, *p* < 0.01). Given slight skewness in the data, a Spearman correlation was also computed, confirming the robustness of this result (rho = −0.54, *p* < 0.01). The correlation was not significant for those evaluated by the preadolescents after correcting for multiple comparisons. In other words, preadolescents with high effortful control (as observed by parents) were less sensitive to negative emotions.

The same temperamental trait data were correlated with the individual RT emotion‐conflict score, which yielded a relatively consistent relational pattern as illustrated in Figure 2c,d and summarized in Table 7. Here, negative affect and effortful control, respectively, were negatively and positively correlated to the emotion‐conflict score, that is, participants scoring high in effortful control and low in negative affect showed faster emotion‐conflict processing. Only the correlation between effortful control evaluated by the parents and the emotion‐conflict score was significant (*R* = −0.63, *p* < 0.01; Spearman rho = −0.56, *p* < 0.01). after correcting for multiple comparisons.

**TABLE 7:** Pearson's correlations between effortful control and negative conflict and emotion‐conflict score.

		Effortful control (parents)	Effortful control (preadolescents)	Negative affect (parents)	Negative affect (preadolescents)
RT conflict score	puncorr	0.034^*^	0.09	0.45	0.83
	pcorr	0.10	0.18	0.25	0.83
	*R*	−0.44	−0.40	0.16	−0.05
RT emotion‐conflict score	puncorr	0.001^***^	0.60	0.04*	0.24
	pcorr	0.008^**^	0.69	0.11	0.32
	*R*	−0.63	−0.12	0.43	0.25

*Note*: puncorr represents the p‐values uncorrected for multiple comparisons; pcorr represents the *p*‐values corrected for multiple comparisons using the Benjamini–Hochberg method.

* *p* ≤ 0.05.

** *p* ≤ 0.01.

*** *p* ≤ 0.001.

### 4.2 fMRI Data

#### 4.2.1 GLM Analysis

**FIGURE 3:**
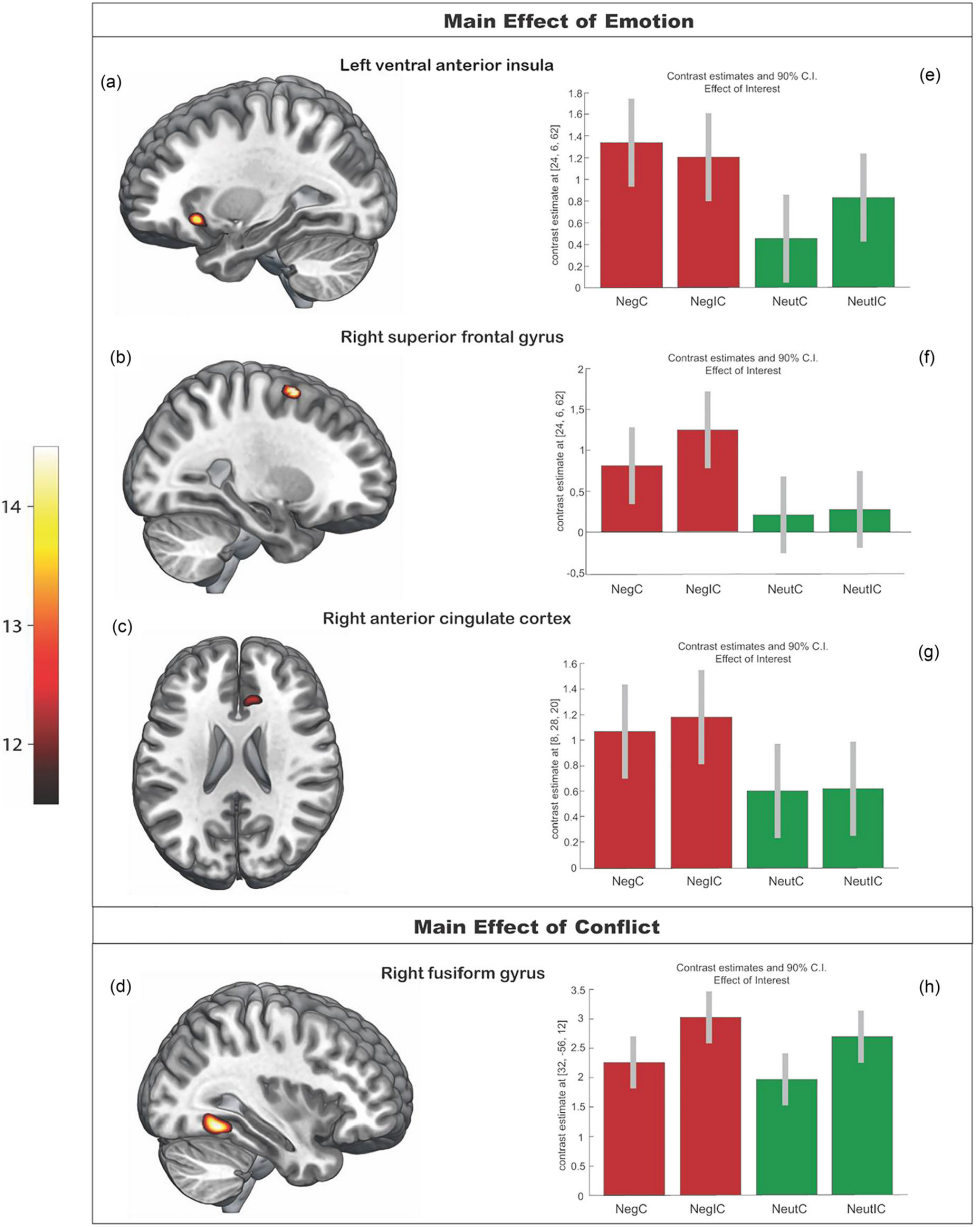
GLM group‐level results for the main effect of EMOTION (a–g) and the main effect of CONFLICT (d, h). Significance thresholds were set at *p* > 0.05 (FWER), using a cluster‐extent‐based thresholding method. Bar graphs show contrast estimates and 90% confidence intervals to determine the direction of the interaction.

The GLM results are shown in Table 8 and Figure 3. The main effect of EMOTION showed increased activity in the left lvAI (Figure 3a), the rSFG (Figure 3b), and the rACC (Figure 3c), whereas a main effect of CONFLICT yielded increased activity in the right fusiform gyrus (rFG; Figure 3d). The direction of the interactions was identical to all regions found for the main effect EMOTION (Figure 3e–g), that is, the activity was consistently greater in negative compared to neutral trials. For the main effect of CONFLICT, the resulting region showed more activity for incongruent than for congruent trials (Figure 3h).

**TABLE 8:** MNI coordinates and local maxima of the resulting regions from GLM full‐factorial analysis (*F*‐contrasts) for the main effects of both EMOTION and CONFLICT.

	Region	Cluster size	MNI coordinates			*F*
			*x*	*y*	*z*	
Main effect EMOTION	Right SFG	50	24	6	62	19.95
	Left vAI	49	−28	22	−8	16.86
	Right ACC	29	8	28	20	13.74
Main effect CONFLICT	Right FG	122	32	−56	−12	18.53
	Right mOG	75	36	−86	8	14.81

*Note*: Cluster size is in voxels.

Abbreviations: ACC = anterior cingulate cortex, FG = fusiform gyrus, mOG = middle occipital gyrus, SFG = superior frontal gyrus, vAI = ventral anterior insula.

#### 4.2.2 Connectivity Analysis

**FIGURE 4:**
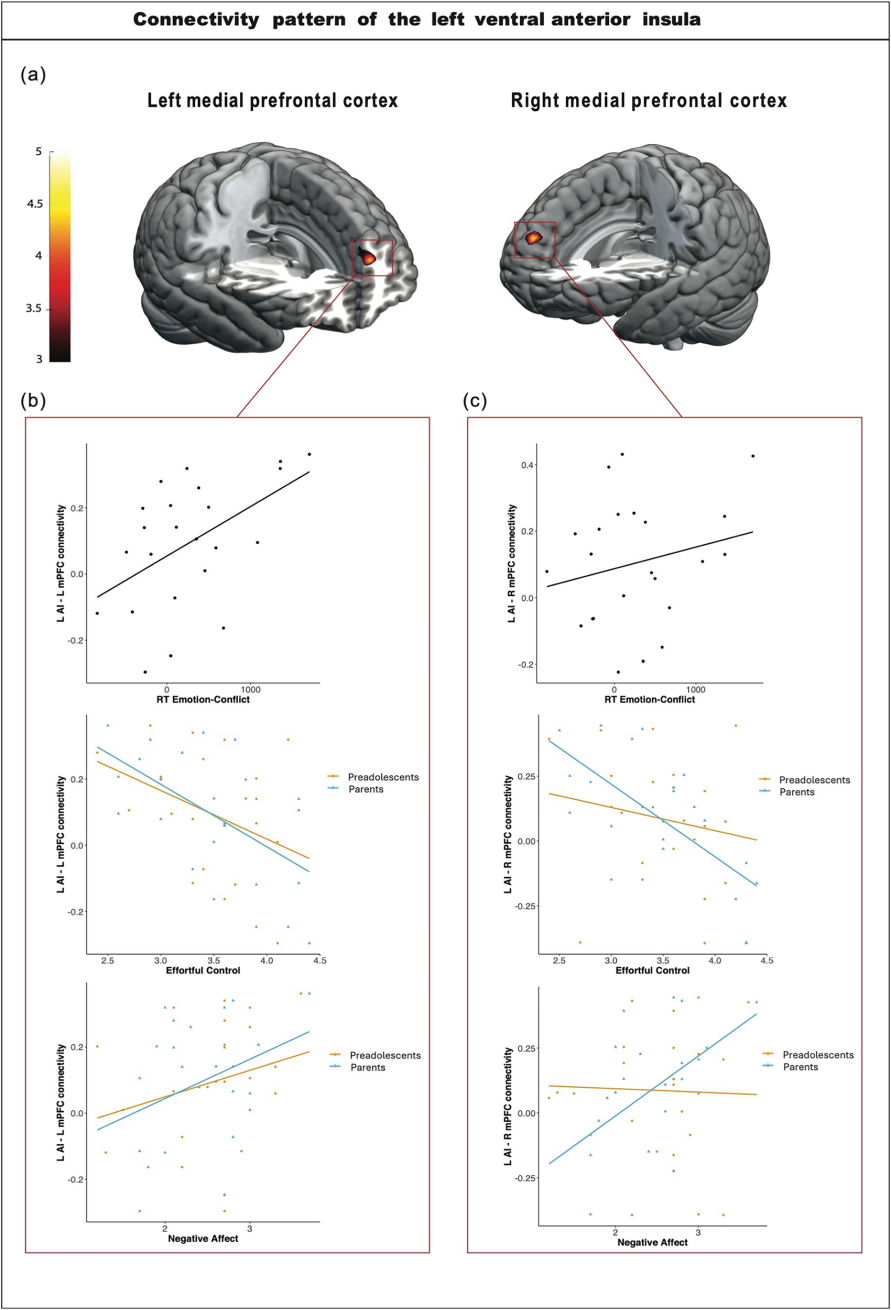
(a) Seed‐based connectivity pattern of the left ventral anterior insula for the main effect of EMOTION. Significance thresholds were set at *p* > 0.05 (FWER), using a cluster‐extent‐based thresholding method. (b, c) Pearson's correlation graphs between behavioral scores (emotion‐conflict score, effortful control, and negative affect) and the resulting connectivity pattern of the left anterior ventral insula. L mPFC = left medial prefrontal cortex, L AI = left (ventral) anterior insula, R mPFC = right medial prefrontal cortex.

We conducted seed‐based functional connectivity analyses for the main effect of EMOTION. No significant changes in functional connectivity involving the bilateral amygdala were observed. The connectivity results for other regions are shown in Tables 7 and 8, and Figure 4. Increased functional connectivity was found between the lvAI and the right medial prefrontal cortex (R mPFC; coordinates: 2, 62, 30, *k* = 38, *F* = 4.87) and left medial prefrontal cortex (L mPFC; coordinates: −8, 54, 22, *k* = 33, *F* = 4.32) for the main effect EMOTION (Figure 4a).

Connectivity increased between the lvAI and bilateral mPFC for the main effect of EMOTION (Figure 4a; Table 9) for all participants. The correlation between behavioral results and lvAI connectivity patterns is shown in Figure 4b,c and Table 10. For the main effect of EMOTION, the connectivity between the lvAI and bilateral mPFC significantly decreased in participants with high effortful control, according to the parents. There were no significant correlations between this connectivity pattern and negative affect, as well as the RT emotion‐conflict score. However, there was a tendency for increased connectivity in participants with high sensitivity to negative affect rated by the parents and low speed in conflict processing in negative emotional trials.

**TABLE 9:** MNI coordinates and local maxima of the resulting regions from the GLM full‐factorial analysis (*F*‐contrasts) for the main effects of both EMOTION and CONFLICT.

	ROI	Region	Cluster size	MNI coordinates			*t‐value*
Main effect of EMOTION	Left vAI			***x***	***y***	***z***	
		Right mPFC	38	2	62	30	4.87
		Left mPFC	33	−8	54	22	4.32

*Note*: Cluster sizes are in voxels.

Abbreviations: mPFC = medial prefrontal cortex, PCC = posterior cingulate gyrus, vAI = ventral anterior insula.

**TABLE 10:** Pearson's correlations between conflict score and emotion‐conflict score with the resulting seed‐based connectivity analysis from the left ventral insula for the main effect of EMOTION.

	Effortful control (parents)		Effortful control (preadolescents)	Negative affect (parents)	Negative affect (preadolescents)	RT emotion‐conflict score	RT conflict score
R mPFC	puncorr	0.00034***	0.42	0.012**	0.88	0.17	0.50
	pcorr	0.0017***	1	0.06	1	0.83	0.50
	*R*	−0.68	−0.18	0.51	−0.03	0.30	0.14
L mPFC	puncorr	0.0033***	0.08	0.12	0.23	0.01**	0.18
	pcorr	0.0017***	0.42	0.60	1	0.07	0.36
	*R*	−0.58	−0.37	0.33	0.30	0.50	0.28

*Note*: pcorr represents the p‐values FDR‐corrected. puncorr represents the *p*‐values uncorrected for multiple comparisons.

Abbreviations: L mPFC = left medial prefrontal cortex, R mPFC = right medial prefrontal cortex.

**p ≤ 0.01.

***p ≤ 0.001.

## 5 Discussion

In the present study, we aimed to elucidate the neural underpinnings of processes underlying an emotion–cognition interaction and their relationship with self‐regulation abilities in preadolescents. We used a CFT and a temperament trait questionnaire for the latter abilities. Behavioral results showed that conflict and negative emotion each significantly and independently prolonged RTs. Descriptively, the longest response times were observed when conflict and negative emotion occurred together, although including their interaction did not improve model fit, suggesting no added explanatory value over the main effects model. This indicates that negative emotion did not significantly modulate the effect of conflict. The processing of negative words relative to neutral words was accompanied by increased activity in the left vAI, right SFG, and right ACC, but also with higher functional connectivity between the mPFC and left vAI. Correlations between individual temperament trait, emotion‐conflict score, and connectivity strength revealed that preadolescents with low self‐regulation abilities (poor effortful control and high sensitivity to negative stimuli) had a lower ability to resolve conflict associated with stronger mPFC–vAI connectivity while processing negative words.

Although the RT results are consistent with previous studies reporting impaired cognitive control in an emotional context (Hare et al. 2008; Dreyfuss et al. 2014), the current study implies rather more prudent behavior than an impulsive response to negative stimuli in preadolescents. In the case of perceived aversive stimuli, the defensive motivation system is triggered to allow the individual to prepare for action. The person can then increase her/his effort to resolve blockage (i.e., fight), abandon efforts toward a lost target (i.e., flight), or exhibit an immobile and attentive behavior, more thought of as an intermediate step to mobilize the organism to make a decision for an appropriate action (Mobbs et al. 2015). It has also been proposed that seeing pictures of negative valence induces focused attention and motor inhibition, usually expressed with slower response times (Sagaspe et al. 2011), comparable to animal freezing (Sagliano et al. 2014). Therefore, a plausible explanation for the present finding could be that viewing negative words generates a freezing‐like behavior, slowing the response times, especially in a conflict situation that may require more time to process both emotional and cognitive dimensions. As we did not find differences in accuracy across conditions, we suggest that words of negative emotion increased the competition for cognitive resources and interfered with conflict processing. This result is consistent with recent findings reporting slower responses to fear than happy or calm expressions, which were higher for adolescents than adults and suggested to reflect heightened bottom‐up processes in adolescence (Bos et al. 2020). Although with a small effect size, comparable results were found in a recent study with preadolescents (10–11) where angry words prolonged RT relative to happy words using an emotional word‐emotional face stroop (Smolker et al. 2022). In a similar vein, adults showed opposite reactions in the same CFT with faster responses to negative words; this implies that negative trials speeded up conflict processing (Kanske and Kotz 2011, 2012). According to the authors, this behavior might reflect an adaptive mechanism where emotional situations trigger an effective attentional process, allowing the rapid detection and response to a potential threat.

In adults, the faster response to negative words was supported by activation in the ACC and the amygdala, which, together with the mPFC, are typically involved in downregulatory processes and emotional interference (Etkin et al. 2011). Here, preadolescents exhibited enhanced activation in affect (left vAI, right ACC) and cognitive areas (right SFG) for negative rather than neutral words, but not the amygdala. Two explanations might explain this result. First, despite the finding that emotional pictures and words may elicit similar emotional reactions (Schacht and Sommer 2009; Schlochtermeier et al. 2013), words may be less arousing (Hinojosa et al. 2009), given that language requires lexico‐semantic processing to understand meaningful concepts. It has also been shown that while emotion language comprehension (i.e., knowing the meaning of emotional words) appears to plateau in preadolescence, emotion concept abstraction (i.e., representing emotions in terms of psychological internal states), which is crucial for the language‐related experience of emotion, continues to develop into adulthood (Nook et al. 2019). Thus, the lack of amygdala activation in the present study may be due to preadolescents being less aroused in response to words than pictures or faces commonly used in adolescent studies (Hare et al. 2008; Gee et al. 2013; Raschle et al. 2017; Silvers et al. 2017). It could also be due to the ongoing development of the association between understanding emotional words and experience between childhood and adulthood. Nonetheless, we found activation in the ACC and the vAI, which are functionally connected, together with other limbic regions including the amygdala. The vAI is part of the paralimbic system and involved in emotion processing (Eisenberger 2015; Uddin et al. 2017), but also in cognitive–emotion interactions (Smith et al. 2014; Raschle et al. 2017) and may be essential for the successful regulation of executive control. The right SFG has recently been associated with inhibitory control and less motor urgency (Hu et al. 2016); therefore, its activation may be due to the inhibition of the response for the negative words to successfully resolve conflict in an emotional context.

To better understand the slower responses to negative words, we focused on the main effect of emotion (negative vs. neutral words contrast) and explored functional connectivity between regions identified in the GLM analysis (i.e., left vAI, right SFG, and right ACC) as ROIs. Only the left vAI exhibited significant connectivity differences in this condition, with higher connectivity with bilateral mPFC. Interestingly, several findings from neuroimaging studies and experimental lesion studies in humans and rodents emphasize the importance of the ventral mPFC and its top‐down inhibition of the amygdala and vAI in the control of negative emotions (Coombs et al. 2014; Motzkin et al. 2015), with an earlier development of ascending projections (limbic‐prefrontal) than descending projections (prefrontal‐limbic) (Bouwmeester et al. 2002; Cressman et al. 2010). As discussed earlier, preadolescence appears to be a critical period for shifts from subcortical‐cortical to cortical‐subcortical connectivity and the prefrontal‐limbic connectivity pattern, which is essential for effective emotion regulation, seems to be mature by late adolescence. Although reduced fronto‐limbic connectivity may seem counterintuitive, several studies suggest it reflects a normative maturational shift toward more specialized and efficient emotion regulation. Rather than indicating under‐engagement, decreased coupling (such as between the mPFC and vAI) has been linked to the pruning of diffuse connections and increased selectivity in top‐down control as adolescents rely less on constant regulatory input to manage affective responses (Baum et al. 2017; Jones et al. 2023). This is consistent with developmental findings showing a shift from positive to negative functional connectivity between the amygdala and prefrontal regions, particularly the mPFC, during the transition from childhood to adolescence (Gee et al. 2013; Wu et al. 2016). Therefore, the heightened functional connectivity between the mPFC and the vAI, together with increased RTs for negative stimuli, may reflect this developmental transition with immature top‐down connections, resulting in greater recruitment of the mPFC necessary to downregulate the vAI reactivity to emotional words.

Given the huge interindividual differences in RTs, we examined individual self‐regulation traits and found that both effortful control and negative affect were related to the emotion‐conflict score (reduced scores corresponded to faster conflict resolution in an emotionally salient context) and vAI–mPFC connectivity strength. For those correlations with individual traits, we only found that effortful control evaluated by the parents reached significance despite consistent tendencies when assessed by the preadolescents. This could be explained by a lower ability to evaluate themselves relative to their parents, with overtly positive or negative self‐judgments (Pfeifer and Berkman 2017).

Results show that individuals high in effortful control generally exhibited lower negative affect, which is in line with previous results showing that higher cognitive abilities allow for more efficient processing of cues evoking stress or threat and facilitate goal‐oriented behaviors during preadolescence (Thompson et al. 2014; Lehikoinen et al. 2019). This assumption is also corroborated by the relationship we found between faster emotion‐conflict processing and both higher effortful control and lower negative affect. Indeed, it suggests that individuals with a tendency to experience a higher intensity of negative emotional reactions, together with difficulties in inhibiting their responses and focusing their attention, display a greater interference of emotional words with conflict processing. This lower capacity to move away from emotional content to focus on cognitive performance is thought to lead to maladaptive regulation of emotions (especially rumination and self‐blame) and is linked to higher vulnerability for depression and anxiety (see Schäfer et al. [2017] for meta‐analysis). Interestingly, effortful control was negatively correlated with vAI–mPFC functional connectivity, in contrast to negative affect, which was positively correlated. As reported above, individuals low in effortful control also displayed higher negative affect, indicating that preadolescents with poorer cognitive abilities and higher sensitivity toward negative stimuli exhibited stronger functional coupling between the vAI and mPFC during conflict resolution in an emotional context. This may be due to greater recruitment of the mPFC to downregulate the vAI reactivity, mirroring recent results showing that high negative affect and low cognitive control predict a less mature pattern of PFC–amygdala connectivity (stronger connectivity) in emotion regulation (Davis et al. 2019). Similarly, the greater vAI–mPFC coupling found in individuals with lower self‐regulation abilities may be due to an altered or less developed pattern of connectivity between these areas, resulting in less effective conflict resolution in emotionally salient contexts. Supporting this idea, greater AI–mPFC connectivity and exaggerated left AI and mPFC activation were previously associated with increased depression severity in adults and adolescents (Beauregard et al. 2006; Kaiser et al. 2016; Jankowski et al. 2018), and dysfunction of these areas is thought to lead to low disengagement from negative information (Ai et al. 2015). The vAI and mPFC are key regions involved in the integration of interoceptive and affective information (Craig 2009; Li et al. 2017), and their functional connectivity may reflect individual differences in emotion regulation abilities. This interpretation aligns with developmental evidence from studies using emotional faces and pictures, which show that key emotion regulation networks (including amygdala–prefrontal and insula–prefrontal pathways) undergo protracted maturation from preadolescence into adolescence (Hare et al. 2008; Gee et al. 2013; Tottenham and Galván 2016; Silvers et al. 2017).

In this context, our findings suggest that preadolescents with heightened sensitivity to negative emotions and lower cognitive control may exhibit increased engagement of the vAI–mPFC circuit when processing emotionally salient words, potentially reflecting an immature regulation mechanism that relies on heightened recruitment of frontal control regions. Given the role of the vAI and mPFC in integrating affective and interoceptive information (Craig 2009; Smith et al. 2014), this pattern may index a stronger internal emotional response that is more difficult to disengage from. However, as all participants were still in a phase of ongoing neurodevelopment, these findings should be interpreted with caution and seen as part of a broader maturational trajectory.

## 6 Limitations

Because of practical difficulties in conducting pediatric neuroimaging research, especially with the need to keep preadolescents motivated, we decided to remove words of positive valence and shorten the MRI session. Focusing only on negative words allowed us to increase power and confidence in the analysis by reducing the probability for random responses to occur due to habituation and exhaustion. Moreover, as each word was presented only once, we did not model “item” as a random effect in the RT analysis. While this design choice was appropriate given the absence of item‐level repetition, it may limit the generalizability of the behavioral findings beyond the specific stimulus set used. Finally, our sample size was relatively small, which might have affected the behavioral analysis, limiting the generalization of the results, although such a size reflects the challenges of performing fMRI studies in healthy preadolescents.

## 7 Conclusion and Perspectives

The present study investigated whether individual differences in self‐regulation abilities are associated with abilities to resolve conflict in an emotional context and with neural regulation of emotions in preadolescents. We found that individuals with low self‐regulation abilities, as reflected by high negative affect and low effortful control, are slower to resolve conflict and display enhanced vAI–mPFC functional connectivity while processing negative words relative to neutral words.

These results deliver new insights into the preadolescent's emotion regulation; first, by introducing the coupling vAI–mPFC as a key circuit involved in experiencing emotions expressed in words and regulation in preadolescents, in addition to the classically amygdala–mPFC network investigated using emotional pictures or faces. Second, showing that discrepancies among individuals in self‐regulatory competencies predict both emotion regulation abilities and vAI–mPFC connectivity suggests that such individual differences may underlie normal temperament variations when facing negative situations. These individual differences also identify the potential associated risk for developing maladaptive strategies during this sensitive period of life. As correlations do not infer causality, future neuroimaging research will need to address the causal mechanisms of these individual behavioral and brain differences and whether they relate to the onset of psychological disorders such as anxiety and depression.

## Author Contributions

**P. Cantou**: conceptualization, data curation, formal analysis, investigation, methodology, visualization, writing – original draft, writing – review and editing. **B. Kleber**: data curation, formal analysis, investigation, methodology, supervision, writing – review and editing. **S. A. Kotz**: conceptualization, data curation, methodology, resources, software, supervision, writing – review and editing. **P. Vuust**: funding acquisition, project administration, resources, supervision, writing – review and editing. **M. C. Fasano**: data curation, investigation, methodology, supervision, writing – review and editing. **E. Brattico**: conceptualization, funding acquisition, investigation, project administration, resources, supervision, writing – review and editing.

## Conflicts of Interest

The authors declare no conflicts of interest.

## Peer Review

The peer review history for this article is available at https://publons.com/publon/10.1002/brb3.70895

## Supporting information




**Supplementary Material**: brb370895‐sup‐0001‐TableS1‐S6.docx

## Data Availability

The data that support the findings of this study are available from the corresponding author upon reasonable request.
