# Supplementary material for: Emotion Regulation in the Preadolescent Brain and the Role of Individual Temperamental Differences
Source: Brain Behav. 2025 Oct 21;15(10):e70895. doi: 10.1002/brb3.70895 (PMC12540916; doi:10.1002/brb3.70895)
Supplement: Supplementary file 1 — Supplementary Material: brb370895‐sup‐0001‐TableS1‐S6.docx [file BRB3-15-e70895-s001.docx]

**Supplementary material**

**Supplementary Table 1.** Participant age, PDS scores, and Puberty Categories (based on Crockett, 1988 criteria).

| **Gender** | **N** | **Mean Age (years)** | **SD Age (years)** | **Mean PDS** | **SD PDS** | **Prepubertal** | **Early pubertal** | **Midpubertal** | **Postpubertal** |
| --- | --- | --- | --- | --- | --- | --- | --- | --- | --- |
| **Female** | 10 | 10.81 | 1.40 | 1.92 | 0.65 | 0 | 2 | 6 | 2 |
| **Male** | 13 | 10.28 | 1.24 | 1.42 | 0.30 | 6 | 6 | 1 | 0 |
| **Total** | 23 | 10.54 | 1.32 | 1.63 | 0.54 | 6 | 8 | 7 | 2 |

**Supplementary Table 2.** ANOVA-based likelihood ratio tests of the models for raw reaction time (LMM).

|  | **Df** | **AIC** | **BIC** | **logLik** | **deviance** | **Chisq** | **Chi Df** | **Pr(>Chisq)** |
| --- | --- | --- | --- | --- | --- | --- | --- | --- |
| Model1 | 3 | 33959.57 | 33976.13 | -16976.79 | 33953.57 |  |  |  |
| Model2 | 4 | 33791.72 | 33813.79 | -16891.86 | 33783.72 | 169.86 | 1 | .00 *** |
| **Model3** | 5 | 33693.56 | 33721.15 | -16841.78 | 33683.56 | 100.15 | 1 | .00 *** |
| Model4 | 6 | 33693.88 | 33726.99 | -16840.94 | 33681.88 | 101.83 | 2 | .20 |

Supplementary Table 3. ANOVA-based likelihood ratio tests of the models for log-transformed reaction time (LMM).

|  | Df | AIC | BIC | logLik | deviance | Chisq | Chi Df | Pr(>Chisq) |
| --- | --- | --- | --- | --- | --- | --- | --- | --- |
| Model1 | 3 | -295.00 | -278.44 | 150.50 | -301.00 |  |  |  |
| Model2 | 4 | -505.69 | -483.62 | 256.84 | -513.69 | 212.69 | 1 | .00 *** |
| Model3 | 5 | -601.17 | -573.59 | 305.59 | -611.17 | 97.49 | 1 | .00 *** |
| Model4 | 6 | -599.21 | -566.10 | 305.61 | -611.21 | 0.03 | 2 | .85 |

Supplementary Table 4. Linear mixed model fit by maximum likelihood of the fixed and random effects of Model3 for log-transformed reaction time (LMM).

| FIXED EFFECTS | | | |
| --- | --- | --- | --- |
|  | ESTIMATE | SE | T-value |
| (Intercept) | 9.07 | 0.06 | 153.33 |
| CONFLICT (Incongruent) | 0.14 | 0.01 | 15.43 |
| EMOTION (Negative) | 0.09 | 0.01 | 10.01 |
| RANDOM EFFECTS | | | |
| GROUPS | NAME | VARIANCE | SD |
| PARTICIPANTS | (Intercept) | 0.08 | 0.28 |
| RESIDUAL |  | 0.04 | 0.20 |

**Supplementary Table 5.** ANOVA-based likelihood ratio tests of the models for accuracy (GLMM).

|  | **Df** | **AIC** | **BIC** | **logLik** | **deviance** | **Chisq** | **Chi Df** | **Pr(>Chisq)** |
| --- | --- | --- | --- | --- | --- | --- | --- | --- |
| Model 1 | 2 | 1166.0 | 1177.0 | -580.98 | 1162.0 |  |  |  |
| Model 2 | 3 | 1167.6 | 1184.1 | -580.78 | 1161.6 | 0.39 | 1 | .533 |
| Model 3 | 4 | 1166.1 | 1188.1 | -579.03 | 1158.1 | 3.50 | 1 | .061 |
| Model 4 | 5 | 1165.4 | 1193.0 | -577.71 | 1155.4 | 2.64 | 1 | .104 |

**Supplementary Table 6.** Least squares mean comparisons for model3 for accuracy. P-values are adjusted using Bonferroni correction.

| Contrasts | **Estimate** | **SE** | **z value** | **P-value** |
| --- | --- | --- | --- | --- |
| Negative Congruent – Neutral Congruent | –0.047 | 0.214 | –0.22 | 1.000 |
| Negative Congruent – Negative Incongruent | 0.134 | 0.208 | 0.64 | 1.000 |
| Negative Congruent – Neutral Incongruent | –0.424 | 0.229 | –1.85 | 0.383 |
| Neutral Congruent – Negative Incongruent | 0.181 | 0.210 | 0.86 | 1.000 |
| Neutral Congruent – Neutral Incongruent | –0.377 | 0.230 | –1.64 | 0.610 |
| Negative Incongruent – Neutral Incongruent | –0.558 | 0.225 | –2.48 | 0.079 |
